# Spontaneous Coronary Artery Dissection in a Healthy Male: A Case Report and Literature Review

**DOI:** 10.7759/cureus.7568

**Published:** 2020-04-07

**Authors:** Ramy Abdelmaseih, Mustajab Hasan, Jay Patel, Alauddin Elhag, Jigar Patel

**Affiliations:** 1 Internal Medicine, Ocala Regional Medical Center/University of Central Florida College of Medicine, Ocala, USA; 2 Cardiology, Ocala Regional Medical Center, Ocala, USA

**Keywords:** spontaneous coronary artery dissection, sudden cardiac death, myocardial infarction, coronary artery disease

## Abstract

Spontaneous coronary artery dissection (SCAD) is a rare presentation of acute coronary syndrome (ACS) and can potentially lead to sudden cardiac death. SCAD is particularly seen in young females and/or patients with relatively few cardiovascular risk factors which further makes it a diagnostic conundrum. This article aims to highlight the causes, clinical presentation, treatment options, and complications of SCAD in addition to a case report of a young gentleman who was found to have SCAD.

## Introduction

Spontaneous coronary artery dissection (SCAD) is a rare nonatherosclerotic phenomenon that has emerged as an important cause of cardiovascular events including acute coronary syndrome (ACS), myocardial infarctions (MI) and sudden cardiac death (SCD), especially among young females and cohorts with no or few atherosclerotic or cardiovascular risk factors.

SCAD is defined as a sudden nonatherosclerotic, nontraumatic noniatrogenic disruption or separation of the inner intimal lining of the coronary artery wall, creating an intimal flap or intramural hematoma that compromises the blood flow resulting in myocardial ischemia or MI. The triggering events remain poorly understood but are thought to be either an intimal tear or vasa vasorum bleeding driving the intramural hematoma [1].

It was first reported in 1931 during the post-mortem examination of a 42-year-old female and was described as a right coronary artery (RCA) dissecting aneurysm with atheroma that ruptured during a violent retching attack and led to SCD [2]. Since then and particularly in the last 10 years, the Canadian SCAD study (CanSCAD) [2-6] the largest prospective observational SCAD study -- shed light on this phenomenon and provided us with much knowledge about its natural history, associations, treatment strategies, and long-term cardiovascular events. Nowadays, SCAD has been increasingly recognized with more reported cases due to the readiness and ease of new coronary artery diagnostic tools such as intravascular imaging systems and coronary computed tomography angiography (CCTA) [7] and the earlier use of angiographic investigation in ACS.

The SCAD-associated risk factors include: female sex, pregnancy and postpartum status, multiparity, fibromuscular dysplasia (FMD) and other connective tissue disorders, severe hypertension and illicit drug use, hormonal therapy, and systemic inflammatory diseases. However, many cases have no obvious cause [8].

We report a case of a young gentleman who presented with ACS and was found to have SCAD.

## Case presentation

**Figure 1:**
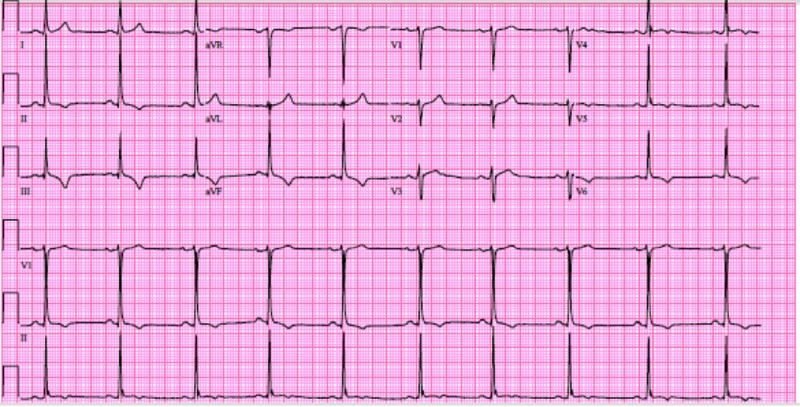
EKG on admission showing T wave inversions in leads II, III, AVF, V4-V6. EKG, electrocardiogram

**Figure 2:**
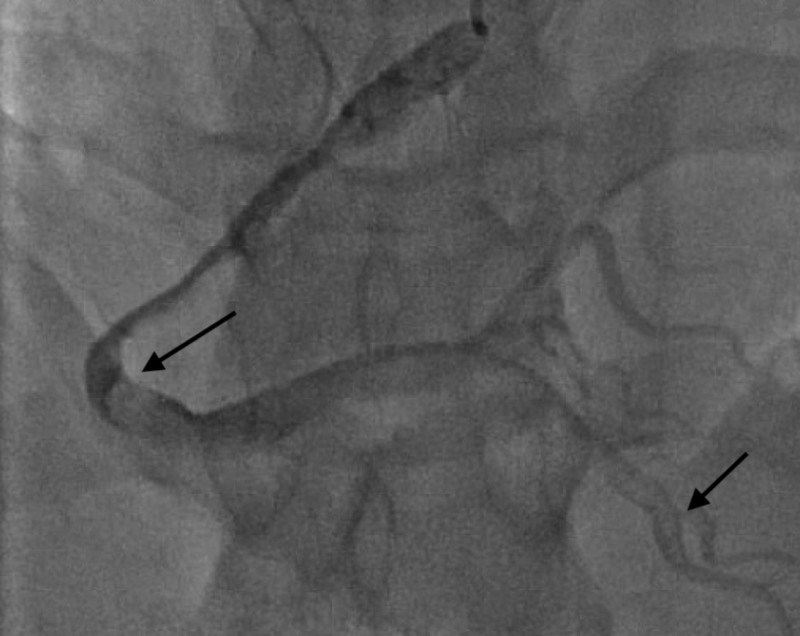
Right coronary vessel showing a proximal vessel lesion (arrow on left) in addition to a right posterior descending artery dissection (arrow on right).

A 34-year-old Caucasian gentleman with no significant past medical history presented to the ED with complaints of progressively worsening exertional dyspnea that started over the week prior in addition to substernal, pressure like chest pain that started on the day of admission. The patient was previously physically active with daily weight lifting and cardiovascular endurance training and had never experienced the aforementioned symptoms before. On arrival to the ED, an electrocardiogram (EKG) was done and showed T wave inversions in leads II, III, AVF, V4-V6 (see Figure 1). Labs were unremarkable other than a Troponin-I of 1.64 followed by 1.55 6 h later. The patient was taken for catheterization which revealed a large right coronary artery with a dissection; lesion appearance was suggestive of FMD. Additionally, the right posterior descending artery also showed a dissection at its origin (see Figure 2). The patients’ medications were optimized with aspirin, clopidogrel, metoprolol, and atorvasatin and was instructed to refrain from physical activity to allow adequate healing of the aforementioned dissection.

## Discussion

Background

Coronary artery dissection was first described in 1931 and since then, at least 1000 cases have been reported. It has been defined as the presence of an intramural hematoma that impinges upon the coronary artery lumen resulting in cessation of coronary blood flow [9]. Coronary artery dissection can be classified into primary dissections which occur spontaneously and secondary dissections that occur following an insult; secondary causes include: cardiac catheterization or surgery, chest trauma [10], or as an extension of an aortic root dissection. Secondary causes must be excluded first before a coronary artery dissection is considered spontaneous.

Epidemiology

The true prevalence remains difficult to estimate, primarily because of under-diagnosis of SCAD and under-recognition of its angiographic findings. It is now increasingly recognized especially after the CanSCAD study, due to the increased awareness of such a phenomenon and improvement in coronary artery imaging studies. The prevalence has risen from about (0.2%-1.1%) to (1.7%-4%) of all ACS cases in recent studies, accounting for about 25% of all ACS cases in females <50 years old. SCAD affects primarily middle-aged women -- about 90% of cases -- approximately 30% of them occurs during the peripartum state and is considered the most common cause of pregnancy associated MI (about 43%). SCAD was reported in all major ethnic and racial groups [6, 10-15].

From literature review of SCAD, it appears that the left anterior descending (LAD) artery is the most commonly affected artery especially the mid to distal segments (60%-75%), and its dissections are more common in women. The right coronary artery (RCA) dissections were observed in 20% of cases and are usually involved in men. Left main artery involvement is in about 4% of cases and left circumflex artery is in 4%. Multivessel dissections were also reported in about 9%-23% of cases [11, 13].

Pathogenesis and etiology

The SCAD etiopathology and involvement in ACS remains poorly understood but is distinct from those of ACS caused by atherosclerosis or plaque rupture. It results from vessel wall hematoma in the absence of traumatic or iatrogenic causes; pressure-driven hematoma expansion through blood or clot accumulation leads to propagation of the dissection plane within the false lumen resulting in compression of the real lumen causing myocardial ischemia or infarction. Patients with SCAD have healthy smooth coronary arteries -- without any atheroma or calcifications -- therefore, they tend to have more extensive dissections, with nonaffected arteries appearing smooth and disease-free in cardiac catheterization.

Common reported associations with SCAD include: pregnancy and peripartum due to estrogen and progesterone-related changes that alter normal arterial wall architecture including smooth muscle cell proliferation, alteration of mucopolysaccharide and protein media content, impaired collagen synthesis, and increased cardiac output and total blood volume that in turn augment shear forces on the luminal arterial surface and increase wall stress making the arteries more susceptible to SCAD. Other associations include: emotional and physical stress [4], any condition that can increase intracoronary shear stress such as increased thoraco-abdominal pressure -- first reported case in 1931 was precipitated by a violent retching attack [2] -- and catecholamines, FMD due to arteriopathy and irregular growth of arterial wall cells, connective tissue disorders (Marfan syndrome, Ehlers-Danlos syndrome, systemic lupus erythematosus vasculitis, and Loeys-Dietz syndrome). However, a large number of cases are idiopathic with no underlying cause.

Clinical presentation

SCAD should be suspected and excluded in young patients -- especially females -- presenting with ACS without having the classical cardiovascular risk factors. An urgent coronary angiography should be warranted.

SCAD has a wide array of clinical presentations and severities, from fatigue, headaches, and dizziness to unstable angina presenting with unexpected chest pain, or acute MI -- 50% of SCAD patients initially present as ST-segment elevation MI with elevated troponin levels -- with radiating chest pain associated with dyspnea, nausea, vomiting, and diaphoresis. About 3%-11% of cases present with ventricular arrhythmias, cardiogenic shock, and SCD. However, in rare instances it can be discovered incidentally on CCTA or during cardiac catheterization [16].

Diagnosis

Early cardiac catheterization and coronary angiography remains the cornerstone in SCAD diagnosis [17]. Using intravascular ultrasound (IVUS) and optical coherence tomography (OCT) imaging can provide a very detailed information about the exact location and extent of dissection [18-19]; yet, they carry additional risks including propagation of the original dissection or causing a new iatrogenic dissection. CCTA can be used in low/intermediate risk patients presenting with ACS and in the outpatient settings for follow-up of patients with SCAD.

Angiographically coronary dissections can be graded into three types including: Type 1 (29%) with classical appearance of multiple radiolucent lumens representing a longitudinal filling defect, Type 2 (the most common 60%) which refers to a long diffuse and smooth narrowing -- usually >30 mm in length -- that can vary in severity from mild narrowing to complete obstruction with no response to intracoronary nitrates, and Type 3 (relatively uncommon accounting for 10% only) with focal or tubular stenosis mimicking atherosclerosis and requiring IVUS or OCT to differentiate the cause [4].

Management

There are no current guidelines for SCAD management and its therapeutic approach remains challenging, uncertain, and relies on the immediate angiographic investigation. However, the conservative medical management is still the preferred strategy after the diagnosis is secured especially when there is no evidence of ischemia or hemodynamic instability [16]. A wide range of therapeutic approaches are available and dependent on the clinical and angiographic findings including: conservative medical management, emergency revascularization with percutaneous coronary intervention (PCI) or coronary artery bypass grafting (CABG), fibrinolytic therapy, mechanical support, and cardiac transplantation.

Initial management with the use of dual antiplatelet therapy (DAPT) with aspirin and clopidogrel, heparin and beta-blockers -- to reduce arterial shear stress and risk of recurrence -- to preserve the true lumen patency, prevent any thrombotic occlusions, and facilitate healing. Glycoprotein IIb/IIIa inhibitors such as ticagrelor and prasugrel can also be used, however, there are no data on their role and they could potentially delay healing and absorption of the intramural hematoma and result in dissection extension. Thrombolytics have no role in SCAD management due to a high risk of bleeding and propagation of the intramural hematoma. Statins benefit in SCAD is still unknown.

Patients who present with acute MI with symptoms of ongoing ischemia or hemodynamic instability should be considered for revascularization with PCI or CABG; yet, it is very challenging and carries a high risk of complications or even failure -- success/partial success rates of 70% based on CanSCAD -- because of coronary artery fragility, technical difficulty advancing the guidewire in the true lumen rather than the false one, and avoiding iatrogenic dissection with distal propagation of the intramural hematoma (occurs in 32%) [6, 13]. Other indications for revascularization are: complete vessel occlusion, sustained ventricular arrhythmias, and recurrent chest pain. Single vessel dissections are usually managed with PCI with stenting, while CABG is considered for patients with left main coronary artery dissections, multivessel involvement, or who underwent an unsuccessful PCI or when PCI is not technically feasible. It is worth mentioning that the ratio of emergency CABG to PCI failure is 10%-13% , hence, PCI should be attempted first, if possible, at centers where cardiac surgeries are available [2, 6].

Prognosis

SCAD has an excellent in-hospital survival rate, with in-hospital mortality rate of 4.2% and recurrent in-hospital MI rate of 4.6%, but has a significant risk of future SCAD (10.4%) and recurrent cardiovascular events (MI 16.8%) [6]. Therefore, patients should be educated about the risk of recurrence, and followed up with coronary arteries diagnostic imaging tools such as noninvasive CCTA. At present, there is no definitive treatment to reduce SCAD’s long-term risk.

## Conclusions

While exceptionally uncommon, SCAD should be suspected and excluded in young patients -- especially females -- presenting with ACS without having the classical cardiovascular risk factors. Although SCAD therapeutic approach remains challenging and is reliant on the immediate angiographic investigation, the conservative medical management is still the preferred strategy unless patients have high risk factors. Proper recognition of SCAD associations should warrant screening for other diseases that may affect body vasculature like FMD. Further research is required to better understand the outcome and long-term event rates with SCAD.
